# OLDER AMERICANS ACT PROVISIONS AND SOCIAL DETERMINANTS OF HEALTH: INTERSECTIONS AND IMPACTS

**DOI:** 10.1093/geroni/igac059.295

**Published:** 2022-12-20

**Authors:** Shannon Griffin, Robbie Skinner, Suzanne Kunkel, Elizabeth Blair, Lois Simon, Robert Graham, Traci Wilson, Kristen Hudgins

**Affiliations:** Insight Policy Research, Arlington, Virginia, United States; Insight Policy Research, Arlington, Virginia, United States; Miami University, Oxford, Ohio, United States; USAging, Washington, District of Columbia, United States; Insight Policy Research, Arlington, Virginia, United States; Miami University, Oxford, Ohio, United States; USAging, Washington, District of Columbia, United States; Administration for Community Living, Washington, District of Columbia, United States

## Abstract

Older Americans Act (OAA) programs and services are strongly linked to social determinants of health (SDOH) and integral to addressing social needs among older adults and individuals with disabilities and their families. Supported by the Administration for Community Living, researchers examined the intersection of OAA-funded programs and services under Titles III, VI, and VII and their impacts on SDOH. This study used an integration of extant literature; secondary data; and interviews with ACL office leadership, AAAs, Title VI program staff, and other local service providers to examine existing social needs among older adults, equity prioritization within OAA-funded program development, and impacts of OAA-funded programs and services. Study findings highlighted relationships between ACL and Aging Network entities, best practices for and process challenges of transforming grant funding into recipient services, and OAA-funded program areas for possible future development.

